# Upregulation of nuclear division cycle 80 contributes to therapeutic resistance *via* the promotion of autophagy-related protein-7-dependent autophagy in lung cancer

**DOI:** 10.3389/fphar.2022.985601

**Published:** 2022-08-29

**Authors:** Xi Chen, Qingchun He, Shuangshuang Zeng, Zhijie Xu

**Affiliations:** ^1^ Department of Pharmacy, Xiangya Hospital, Central South University, Changsha, China; ^2^ Department of Emergency, Xiangya Hospital, Central South University, Changsha, China; ^3^ Department of Emergency, Xiangya Changde Hospital, Changde, China; ^4^ Department of Clinical Laboratory, Xiangya Hospital, Central South University, Changsha, China; ^5^ Department of Pathology, Xiangya Hospital, Central South University, Changsha, China; ^6^ Institute for Rational and Safe Medication Practices, National Clinical Research Center for Geriatric Disorders, Xiangya Hospital, Central South University, Changsha, China

**Keywords:** NDC80, lung cancer, autophagy, ATG7, therapeutic target

## Abstract

Lung cancer remains the leading cause of malignant mortality worldwide. Hence, the discovery of novel targets that can improve therapeutic effects in lung cancer patients is an urgent need. In this study, we screened differentially expressed genes using isobaric tags for relative and absolute quantitation (iTRAQ) analysis and datasets from the cancer genome atlas database, and found that nuclear division cycle 80 (NDC80) might act as a novel prognostic indicator of lung cancer. The expression of NDC80 was significantly increased in lung cancer tissues, as compared to normal tissues, and high expression levels of NDC80 were correlated with unfavorable survival rates. Furthermore, an *in vitro* analysis showed that the stable knockdown of NDC80 decreased the cell viability and increased therapeutic sensitivity in two lung cancer cell lines, A549-IRR and H1246-IRR. Moreover, gene set enrichment analysis results showed that NDC80 was enriched in autophagy-related pathways. The downregulation of NDC80 inhibited the formation of autophagosomes, and reduced the expression of autophagy-related proteins such as LC3II, Beclin-1, and p62 in lung cancer cells. To further clarify the role of NDC80 as a downstream regulator of autophagy, we validated autophagic mediators through iTRAQ analysis and real-time polymerase chain reaction arrays. Autophagy-related protein7 (ATG7) was observed to be downregulated after the knockdown of NDC80 in lung cancer cells. Immunohistochemistry assay results revealed that both NDC80 and ATG7 were upregulated in an array of lung adenocarcinoma samples, compared to normal tissues, and the expression of NDC80 was identified to be positively associated with the levels of ATG7. Our findings suggest that NDC80 promotes the development of lung cancer by regulating autophagy, and might serve as a potential target for increasing the therapeutic sensitivity of lung cancer.

## Introduction

As the leading cause of death worldwide, lung cancer is associated with low 5-years survival rates, which range from 4%–17% (Zhou et al., 2019; Ruiz-Cordero and Devine, 2020). Lung adenocarcinoma (LUAD) and lung squamous cell carcinoma (LUSC) are the most common subtypes of non-small lung cancer (NSCLC) and account for approximately 80%–85% of all cases (Yan et al., 2018; Alexander et al., 2020). Radiotherapy is one of the most effective approaches against lung cancer and is essential for the treatment of all stages of lung cancer through definitive or palliative treatment(Wei et al., 2019; Zhao et al., 2022). However, the advanced intrinsic resistance of lung cancer cells to ionizing radiation (IR) is a severe and frequently observed limitation in patients undergoing radiotherapy. It is critical to explore the mechanisms underlying radioresistance, to enhance treatment efficacy in lung cancer patients.

Autophagy, a process of intracellular catabolic self-digestion, involves the sequestration of dysfunctional proteins and damaged organelles within autophagosomes and the disposal of these components in lysosomes (Li et al., 2020). Recently, emerging studies hav\e shown that radiation therapy can generate cellular stress, which induces autophagy with distinct functions in tumor cells. Autophagy could eliminate the misfolded proteins resulting from IR-induced endoplasmic reticulum stress and is considered to have a protective effect against radioresistance development in various types of cancer cells (Gao et al., 2020). For instance, membrane protein 1 was identified to competitively inhibit the interaction of Bcl-2 with Beclin1, thus increasing autophagy and cell survival after radiation treatment in nasopharyngeal carcinoma (Xu et al., 2021). In hepatocellular carcinoma cells, the knockdown of long non-coding RNA NEAT1 sensitized cells to IR via the downregulation of autophagy-related protein GABARAP (Sakaguchi et al., 2022). Autophagy may stimulate resistance to IR in lung cancer cells by moderating ROS under hypoxic conditions (Chen et al., 2017). Moreover, we had previously confirmed that caveolin-1 could confer IR resistance to NSCLC cells through M-protein-regulated autophagy in the GTPase family, which has immune-related functions (Chen et al., 2021). Even though various studies have reported the role of pro-survival autophagy on radioresistance, the underlying regulatory mechanism remains complex and still needs to be elucidated further.

Nuclear division cycle 80 (NDC80/Hec1), a subunit of a kinetochore complex (also called the NDC80 complex), constitutes and stabilizes microtubule-kinetochore attachment during the segregation of mitotic chromosomes (Sarangapani et al., 2021). NDC80 is comprised of an N-terminal microtubule-binding domain and a C-terminal domain that interacts with other components of the kinetochore complex (Wimbish and DeLuca, 2020). In recent times, interest has been focused on the role of NDC80 in tumor progression. The overexpression of NDC80 has been identified to be an oncogenic biomarker with poor prognosis in several cancers, including gastric and ovarian cancer, and osteosarcoma (Mo et al., 2013; Qu et al., 2014; Xu et al., 2017). Furthermore, the turnover of NDC80 is indispensable for maintaining the conditions necessary for meiosis, in which the loss of phosphorylation of NDC80 at ser-55 and ser-69 causes an erroneous kinetochore-microtubule interaction in colon, lung, and prostate cancers (Chen et al., 2020; Iemura et al., 2021). With regard to cancer treatment, NDC80 might become a novel target for increasing the sensitivity of pemigatinib used for the treatment of cholangiocarcinoma (Scheiter et al., 2021). Several reports obtained using bioinformatic analysis have shown that high expression levels of NDC80 resulted in poor survival in lung cancer patients (Sun et al., 2020; Gao et al., 2022). However, the regulatory effects of NDC80 on progression and radiotherapy efficacy in lung cancer still need to be clarified.

In the present study, we screened NDC80 as a potential biomarker involved in radioresistance development in lung cancer cells using isobaric tags for relative and absolute quantitation (iTRAQ) analysis and the cancer genome atlas (TCGA) database. We confirmed that NDC80 is expressed at high levels in the LUAD and LUSC samples, and is associated with poor prognosis in lung cancer patients. To further analyze the role of NDC80 in radioresistance of lung cancer, *in vitro* studies were performed to show that the knockdown of NDC80 inhibits the proliferation of cells, increases IR sensitivity, and reduces autophagy in lung cancer. Moreover, the upregulation of NDC80 promotes autophagy as it mediates the expression of autophagy-related protein7 (ATG7) in IR-resistant cells. Our data provide a novel prospect of using NDC80 for the regulation of radioresistance and suggest that NDC80 could be used as a novel therapeutic target for improving the sensitivity toward radiation therapy in lung cancer.

## Materials and methods

### Screening of DEGs in LUAD and lung squamous cell carcinoma using iTRAQ analysis and TCGA datasets

Isobaric tags for relative and absolute quantitation (iTRAQ) is a method used for the verification and quantification of proteins *via* quantitative mass spectrometry (Moulder et al., 2018). iTRAQ is performed to identify differentially expressed proteins in A549 parental and IR-resistant cells using reagents from BGI Genomics (Guangzhou, China). The cancer genome atlas (TCGA) database is an open-access platform for the cataloging and discovery of gene expression and clinical prognosis data. Two expression profiling datasets of LUAD and LUSC were downloaded from the TCGA using GDC Application Programming Interface (https://portal.gdc.cancer.gov/repository). The LUAD dataset includes 406 cancerous and 55 non-neoplastic tissues. The LUSC dataset includes 350 cancerous and 42 non-neoplastic tissues. The differentially expressed genes (DEGs) between lung cancer and healthy specimens were identified based on the cut-off criteria (|logFC|>2.5, *p* < 0.05). Using TCGA datasets, LASSO Cox regression was implemented to construct a prognostic model of lung cancer. The LASSO algorithm was used for the normalization of gene expression profiles and shrinkage was performed with the “glmnet” R package (Friedman et al., 2010).

### Bioinformatic analysis of clinical characteristics and GSEA

The university of Alabama at Birmingham (UALCAN) was applied to acquire mRNA and protein data of lung cancer and healthy tissues from the TCGA and Clinical Proteomic Tumor Analysis Consortium (CPTAC) databases (http://ualcan. path.uab.edu/analysis.html) (Chandrashekar et al., 2022). Moreover, UALCAN was used to examine differential expression across cancer types, and perform subtype analysis of the stage and TP53 status. The Xiantao tool(https://www.xiantao.love/products) is a comprehensive interactive web portal used to perform differential expression, survival, and enrichment analysis in various cancer types (Zhang et al., 2021). We used the XianTao tool to perform receiver operating characteristic (ROC) risk evaluation, survival analysis, univariate and multivariate Cox regression analysis, and construct a Nomogram plot. To further identify the prognostic role of NDC80, time-dependent (3-years, 5-years, and 8-years) ROCs were analyzed using the R package “survivalROC” in TCGA datasets (Friedman et al., 2010). For survival analysis, we divided lung cancer samples into high-and low-expression groups according to their median expression using the R “survival” package (Friedman et al., 2010). Moreover, to determine whether NDC80 plays a role in the autophagy process, we used the XianTao tool to perform enrichment using gene set enrichment analysis (GSEA). Furthermore, the gene expression omnibus (GEO) is an open-source platform that contains data regarding gene expression, chips, and microarrays (http://www.ncbi.nlm.nih.gov/geo). Two expression profiling datasets (GSE102287 and GSE8894) were, downloaded from the GEO database, for the analysis of the relationship between the expression of NDC80 and ATG7 (Lee et al., 2008; Mitchell et al., 2017). Kaplan-Meier Plotter (www.kmplot.com) is an open-access database used for the storage of gene expression data and survival information of lung cancer patients. The Kaplan-Meier Plotter was used to analyze the correlation between ATG7 expression and the survival of patients with lung cancer.

### Cell cultures

The parental and IR-resistant NSCLC cell lines, including the LUAD (A549-P/A549-IRR) and LUSC (H1246-P/H1246-IRR) cell lines, were obtained from the Cancer Research Institute, Central South University, China. Parental and IR-resistant cells were cultured using 1,640 medium (8122374, Gibco™, United States) supplemented with 10% fetal bovine serum (04-001-1A/B, BioInd, Israel) and 1% penicillin and streptomycin under 37°C aseptic conditions in the presence of 5% CO2.

### NDC80 shRNA knockdown

The two lentiviral short hairpin RNAs (shRNAs) targeting NDC80 (shNDC80#1, 5′-CAA​GGA​CCC​GAG​ACC​ACT​TAA-3'; shNDC80#2, 5′-GAA​TTG​CAG​CAG​ACT​ATT​AAT-3′) were custom synthesized by Sangon Biotech (Shanghai, China). Each lentiviral NDC80 shRNA was added to cultured IR-resistant cells for 48 h. Stable cells with the NDC80 shRNA were selected using puromycin (10 μg/ml, Sangon Biotech) for a total of 10 days. A scrambled shRNA purchased from Sigma-Aldrich (St. Louis, United States) was used for treating control cells. The protein expression of NDC80 was detected *via* Western blotting.

### RNA extraction and quantitative PCR

The total RNA sequences extracted from NSCLC cells were lysed with TRIzol reagent and then converted to cDNAs using a PrimeScriptTM RT reagent kit (6,210, Takara, Japan). The qPCR assay was performed using the iTaqTM Universal SYBR green Supermix (1725121, Bio-Rad, United States), and β-actin was chosen as an internal control. The sequences of the forward and reverse primers are provided in Supplementary Table S1. The relative expression levels were examined using the 2-ΔΔCT method, as shown in previous reports (Lu et al., 2022), and all the detection-related processes were performed at least three times.

### Western blot analysis

NSCLC cells were collected and lysed using IP lysis buffer with protease inhibitor cocktails (B14012, Bimake, United States) at a ratio of 1:100. Equal amounts of 50 µg lysate samples were loaded onto 10% or 12% SDS-PAGE, and then transferred to PVDF membranes (0.22 µm: ISEQ00010; 0.45 µm: IPVH00010). Membranes were blocked with 5% skimmed milk for 1 h at room temperature, and incubated with primary antibodies diluted in 5% Bovine Serum Albumin (D620272, Sangon Biotech, China) overnight at 4°C. Primary antibodies included the NDC80 (1:1,000;18932-1-AP, Proteintech, China), ATG7 (1:1,000; 10088-2-AP, Proteintech, China), LC3 A/B (1:1,000; 4108S, Cell Signaling Technology, USA), Beclin-1 (1:1,000; 3495S, Cell Signaling Technology, United States), p62 (1:500; sc-28359, Santa, United States) and β-actin (1:2000; sc-58673, Santa, United States) antibodies. Specific bands on membranes were visualized using Immobilon Western chemiluminescent reagents (WBKLS0500, Millipore, United States).

### CCK-8 assays

As reported in a previous study (Xu et al., 2018), cell viability was assessed using the CCK-8 assay. Summarily, cells were digested and seeded into 96-well plates (1 × 10^3^ cells per well). After they were incubated for 24 h, cells were treated with different doses of IR using a gamma irradiator. Subsequently, the CCK-8 test solution (B34304, Bimake, United States) was added for 1 h at 37°C. The optical density (OD) at 450 nm was measured using a spectrometer.

### Colony formation assay

As described in our previous studies (Cui et al., 2019), IR-resistant cells were resuspended and seeded at a density of 1 × 10^3^ per well in a 6-well dish with a complete medium. Following the incubation of cells for 24 h, IR at different doses was used to provide treatment. After approximately 2 weeks of incubation, cells were fixed and stained with 0.3% w/v crystal violet/methanol for 20 min at room temperature.

### Transmission electron microscopy

Cells were digested and collected into 1.5 ml tubes. Then, 2.5% glutaraldehyde solution was used for cell fixation overnight at ambient temperature before being transited to the transmission electron microscopy (TEM) laboratory at the Pathology Department of Xiangya Hospital, Changsha, China, where cells were processed as described in our previous study (Chen et al., 2021). The cells were washed three times using Millonig’s phosphate buffer and incubated for 1 h in 1% osmium tetroxide. The dehydration of the cells was performed using a graded series of 50%, 70%, and 90% acetone for 10 min for each step. Cells were then incubated two times in 100% acetone for 15 min. The process of resin soaking and embedding was performed using a 1:1 mix of acetone: resin for 12 h using samples, and polymerization was conducted with 100% resin overnight at 37°C. For the resin solidification process, cells were treated with 100% resin to allow polymerization to occur overnight at 37°C, and then incubated for 12 h at 60°C. Subsequently, 50–100 nm ultrathin sections of cells were made with an ultramicrotome and a diamond knife. After 3% uranyl acetate and lead nitrate double staining, the cells were examined and imaged on an electron microscope (HT-7700, Hitachi, Japan).

### Immunohistochemistry

The LUAD tissue array and clinical information regarding samples in the array were obtained from Outdo Biotech (Shanghai, China). The deparaffinization of specimens was performed in xylene and rehydration was performed in a graded series of alcohol solutions, as described previously (Xia et al., 2019). Endogenous peroxidase was blocked using 3% H_2_O_2_ after the completion of the microwave antigen retrieval process. The samples were incubated with the primary antibody against the NDC80 antibody (1:500;18932-1-AP, Proteintech, China) and ATG7 antibody (1:4000; 10088-2-AP, Proteintech, China). Two pathologists examined and differentially quantified the images of the sections. The evaluation of IHC intensity was performed and a score of 0 (negative), 1 (weak brown), 2 (moderate brown), or 3 (strong brown) was assigned, while the extent of staining was evaluated by assigning scores of 0 (≤10%), 1 (11%–25%), 2 (26%–50%), 3 (51%–75%), or 4 (>75%). The final staining score was determined by multiplying the intensity score and extent score, and classified as weakly positive (1–3), positive (4–6), and strongly positive (7–12). All paraffin-embedded specimens were collected in accordance with the ethical standards of the human experimental committee.

### Statistical analysis

All experiments were performed in triplicate. The Student’s t-test was performed to compare the differences between 2 data groups, and ANOVA was used for more than 2 data groups. Univariate and multivariate Cox regression was performed for survival analysis. Data analysis was performed using GraphPad Prism 8 and SPSS 23.0. Significant differences were considered at *, *p* < 0.05; **, *p* < 0.01; ***, *p* < 0.001 for all tests.

## Results

### Identification of NDC80 as a radioresistance-related gene in lung cancer

**FIGURE 1:**
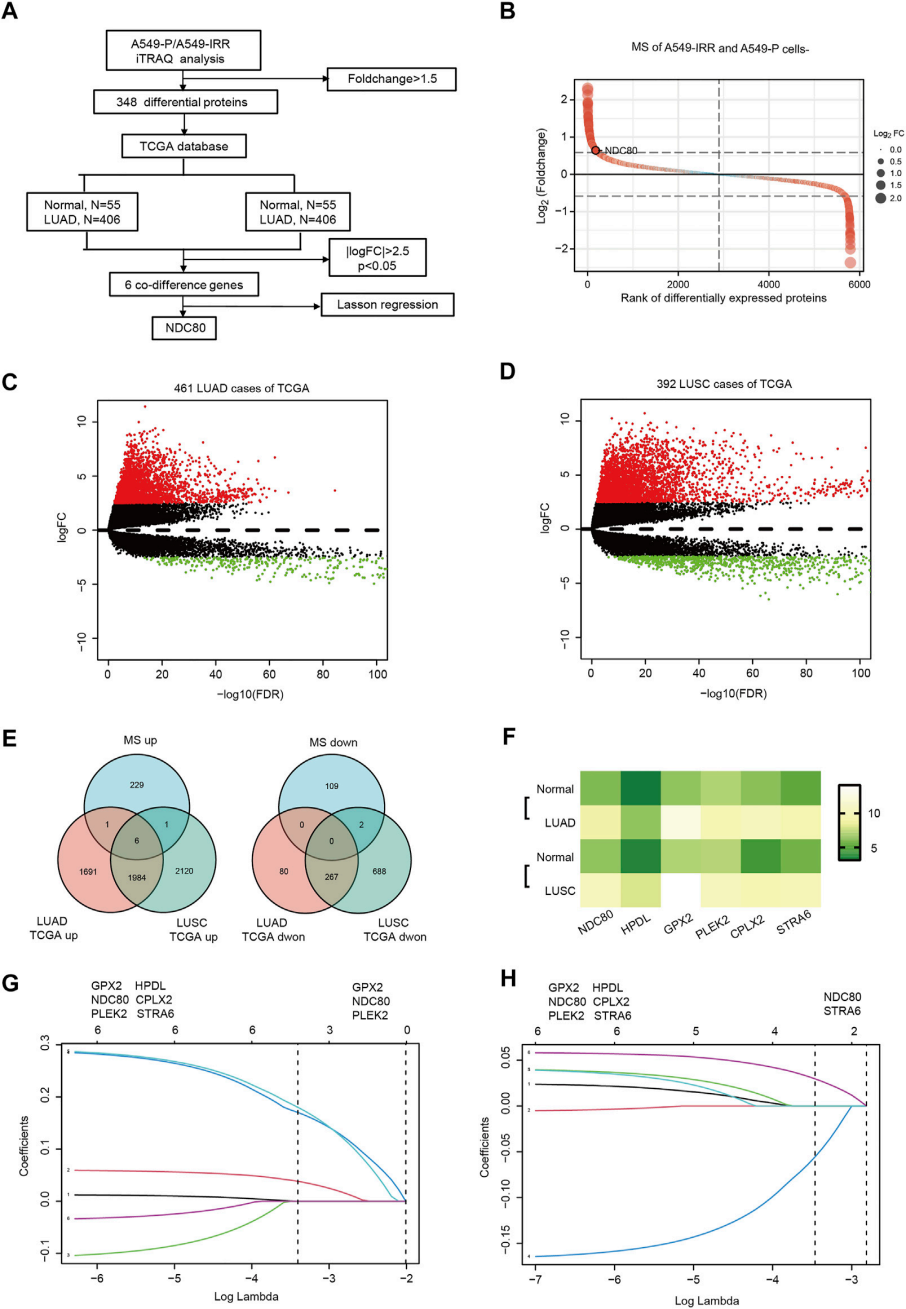
NDC80 is identified as a radioresistance-related gene in lung cancer. **(A)** The flowchart presents the process of identification of genes and their prognostic value in the LUAD and LUSC datasets. **(B)** iTRAQ analysis for the identification of differentially expressed proteins between A549-P and A549-IRR. **(C,D)** Identification of differentially expressed genes in LUAD **(C)** and LUSC **(D)** samples, as compared to paired healthy tissue samples from the TCGA database. **(E)** Visualization of the co-differences in genes from the results of iTRAQ analysis and the TCGA database. The 6 overlapping genes were upregulated in A549-IRR cells and tumor tissues. **(F)** The heatmap shows the expression of 6 overlapping genes in the LUAD and LUSC datasets. **(G,H)** Prognostic genes were identified using the least absolute shrinkage method and selection operator Cox regression model (LASSO) using datasets from TCGA in the LUAD **(G)** and LUSC **(H)** datasets. Coefficient distribution plots for the logarithmic (lambda) sequence for the selection of the best parameter (lambda).

The flow diagram for the present study is shown in Figure 1A. To explore the potential proteins related to radioresistance regulation in lung cancer cells, iTRAQ analysis was performed, to examine the differentially expressed proteins in A549-P and A549-IRR cells. The IR-resistant characteristic of A549-IRR was verified as reported previously (Chen et al., 2021). As shown in Figure 1B and Supplementary Table S2, 5,796 proteins were analyzed, among which 348 proteins were differentially expressed between A549-P and A549-IRR cells (Foldchange>1.5). Two datasets that included 461 LUAD and 392 LUSC samples were selected from the TCGA database. A total of 4029–5,068 DEGs were identified in LUAD and LUSC samples, respectively, as compared to paired non-neoplastic samples (Figures 1C,D, Supplementary Table S3). Combined with information from iTRAQ analysis and two TCGA datasets, 6 co-different genes were identified to be upregulated in lung cancer (Figures 1E,F). Subsequently, we conducted LASSON regression to assess the best fitting variables from the two TCGA datasets. The results suggested that GPX2, NDC80, and PLEK2 represented suitable variables for survival analysis in LUAD datasets (Figure 1G), whereas NDC80 and STRA6 were applicable for use in LUSC datasets (Figure 1H). Intriguingly, only NDC80 was upregulated and related to prognosis in both LUAD and LUSC, and was considered to be the most suitable for further analysis.

### Clinical significance of NDC80 in lung cancer

**FIGURE 2:**
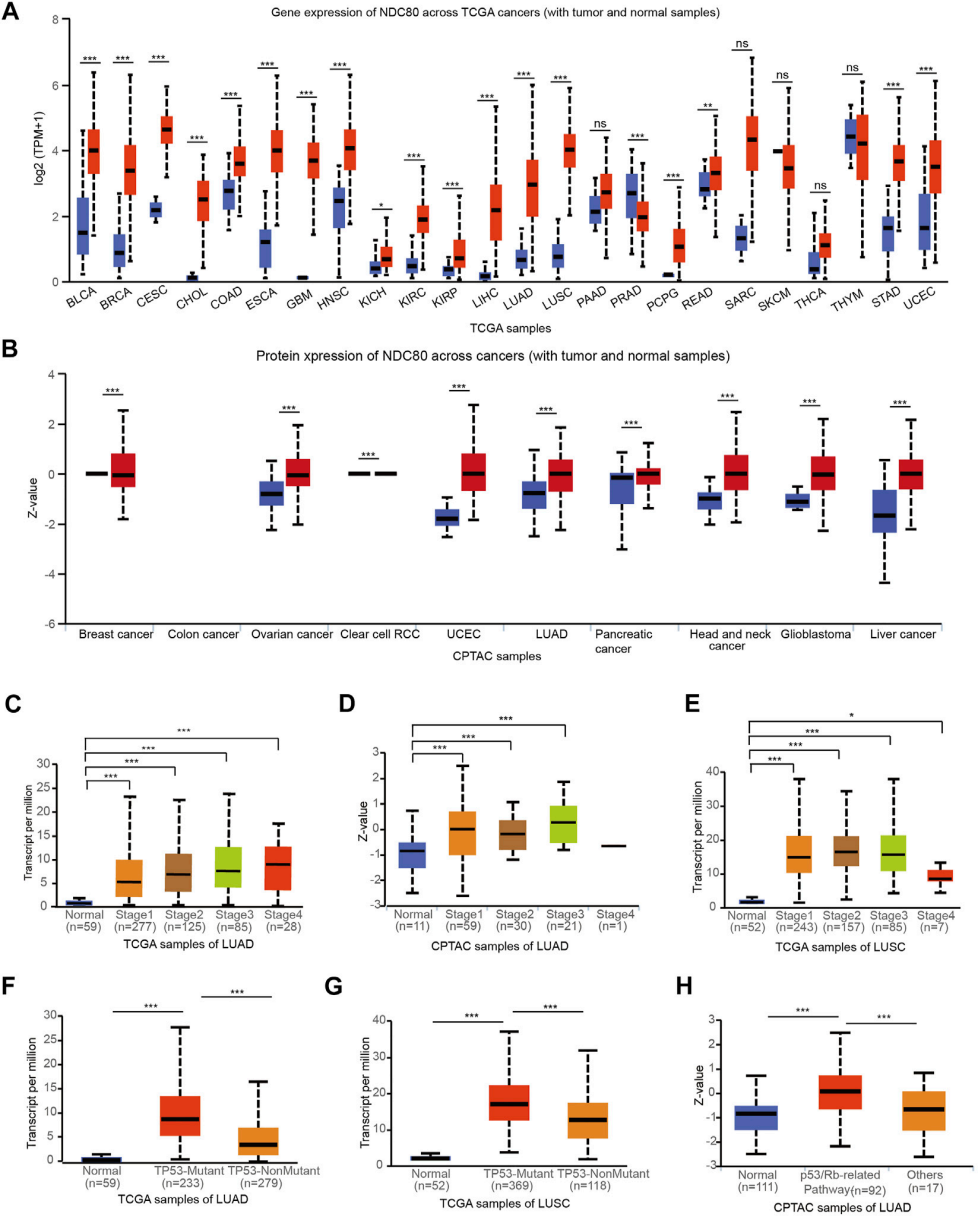
Validation of the expression level and clinical significance of NDC80. **(A,B)** A pan-cancer analysis for the comparison of the transcriptional and protein expression of NDC80 between cancerous and healthy control tissues through the TCGA **(A)** and CPTAC databases **(B)** from UALCAN platforms. **(C–E)** The correlation of the NDC80 expression level with stages of LUAD using the TCGA **(C)** and CPTAC **(D)** databases, and in the LUSC **(E)** dataset. **(F–H)** The association of NDC80 expression level with p53 mutations in the LUAD **(F)** and LUSC **(G)** datasets, and with the p53/Rb pathway in the LUAD **(H)** dataset. *p < 0.05; ***p < 0.001.

To validate the clinical features of NDC80 in the progression of malignancies, pan-cancer analysis was performed using the TCGA and CPTAC datasets from the UALCAN platform. The results obtained using TCGA datasets showed that transcriptional expression levels of NDC80 were higher in 19 types of cancers, as compared to those for matched normal samples (Figure 2A). Meanwhile, the NDC80 expression level was significantly increased in 9 kinds of cancers from CPTAC datasets (Figure 2B). The combined results of pan-cancer analyses showed that the expression of NDC80 was notably elevated in LUAD and LUSC samples. The transcriptional and protein expression of NDC80 was increased in the I-IV and I-III stages of LUAD (Figures 2C,D), while the transcriptional expression of NDC80 was significantly enhanced in stages I-IV of LUSC, as compared to normal tissues (Figure 2E). TP53 mutations are frequent and malignant alterations that are considered unfavorable prognostic biomarkers of lung cancer (Wadowska et al., 2020). We found that transcriptional NDC80 expression was significantly increased in LUAD and LUSC patients with TP53 mutations (Figures 2F,G). In addition, NDC80 was highly expressed in the p53/Rb-related pathways of LUAD patients (Figure 2H).

### Variations in the prognostic value of NDC80 in lung cancer

**FIGURE 3:**
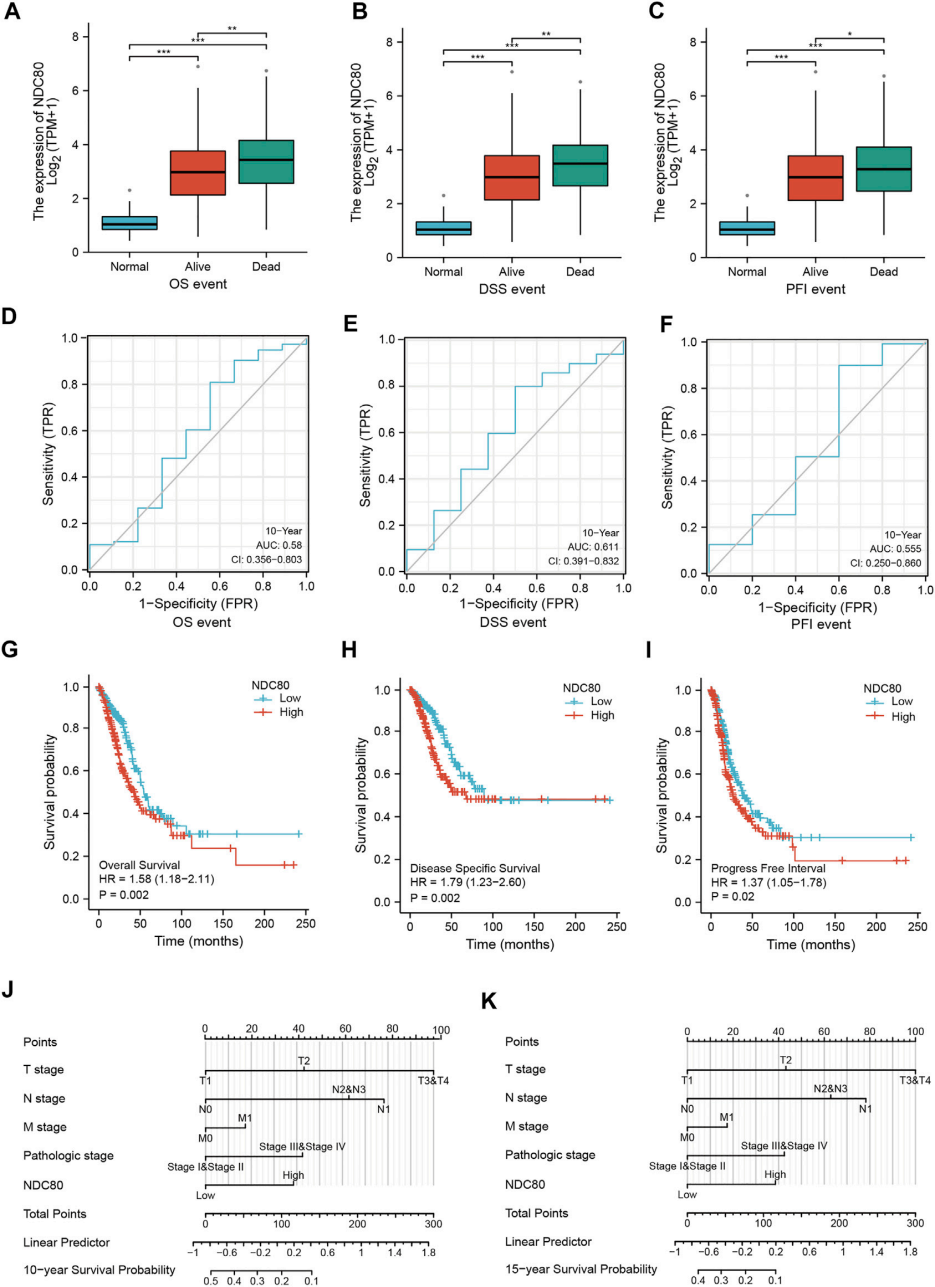
The prognostic value of NDC80 in LUAD. **(A–C)** Using the XianTao tool, the NDC80 expression level was compared among healthy control individuals, and surviving and dead LUAD patients with regard to their OS **(A)**, DSS **(B)**, and PFI **(C)**. **(D–F)** The AUC values of time-dependent ROC curves were used to verify the 10-years prognostic risk score for OS **(D)**, DSS **(E)**, and PFI **(F)**, based on NDC80 expression levels, using the XianTao tool. **(G–I)** Generation of survival curves for the OS **(G)**, DSS **(H)**, and PFI **(I)** of patients in the high-expression and low-expression groups using the XianTao tool. **(J,K)** The prognostic nomogram of LUAD for the 10-years **(J)** and 15-years **(K)** survival period is based on NDC80 expression levels using the XianTao tool. *p < 0.05; **p < 0.01; ***p < 0.001.

To understand the prognostic effect of NDC80 in lung cancer, we performed survival analysis in LUAD patients using the XianTao tool. The highest level of NDC80 was found in the dead patients of LUAD patients during overall survival (OS), disease-specific survival (DSS), and progression-free interval (PFI) events (Figures 3A–C). Moreover, the NDC80-based risk scores were obtained via time-dependent ROC, in which the AUC values for risk score predictions of 10-years OS, DSS, and PFI were 0.580, 0.611, and 0.555, respectively (Figures 3D–F). Similarly, a lower expression level of NDC80 was associated with an improved OS, DDS, and PFI in LUAD patients (Figures 3G–I). Additionally, the results of univariate and multivariate COX analysis also revealed that NDC80 was an independent risk factor of survival in LUAD (Table 1). In the nomogram model, the 10- and 15- year survivals were gradually decreased in patients with advanced TNM stage disease, pathologic stage, and high NDC80 expression levels (Figures 3J,K). Additionally, we further confirmed the prognostic role of NDC80 through datasets downloaded from the TCGA database. A similar tendency was observed for the risk scores of LUAD patients (Supplementary Figures S1A–C). With regard to LUSC patients, we found that the AUCs for patients with a 3-, 5-, and 8-year OS corresponded to 0.721, 0.723, and 0.708 (Supplementary Figures S1D–F). Furthermore, we stratified patients into groups, i.e., the high-risk and low-risk groups, based on variables such as gender, age, and TNM stages. All groups with lower risk scores revealed significantly favorable 3, 5, and 8-years overall survival values, in both LUAD and LUSC patients (Supplementary Figures S1G–L).

**TABLE 1:** The univariate and multivariate Cox regression analysis and clinical features of NDC80.

	Univariate Cox analysis			Multivariate Cox analysis	
Characteristics	Total(N)	HR(95% CI)	*p* value	HR(95% CI)	*p* value
T stage	523				
T1&T2	457	Reference			
T3&T4	66	2.317 (1.591–3.375)	<0.001	2.008 (1.254–3.216)	0.004
N stage	510				
N0	343	Reference			
N1&N2&N3	167	2.601 (1.944–3.480)	<0.001	2.061 (1.401–3.033)	<0.001
M stage	377				
M0	352	Reference			
M1	25	2.136 (1.248–3.653)	0.006	1.275 (0.670–2.426)	0.459
NDC80	526	1.269 (1.128–1.428)	<0.001	1.275 (1.107–1.469)	<0.001
Pathologic stage	518				
Stage I&Stage II	411	Reference			
Stage III&Stage IV	107	2.664 (1.960–3.621)	<0.001	1.288 (0.788–2.105)	0.313

### Knockdown of NDC80 increases the radiosensitivity of lung cancer cells

**FIGURE 4:**
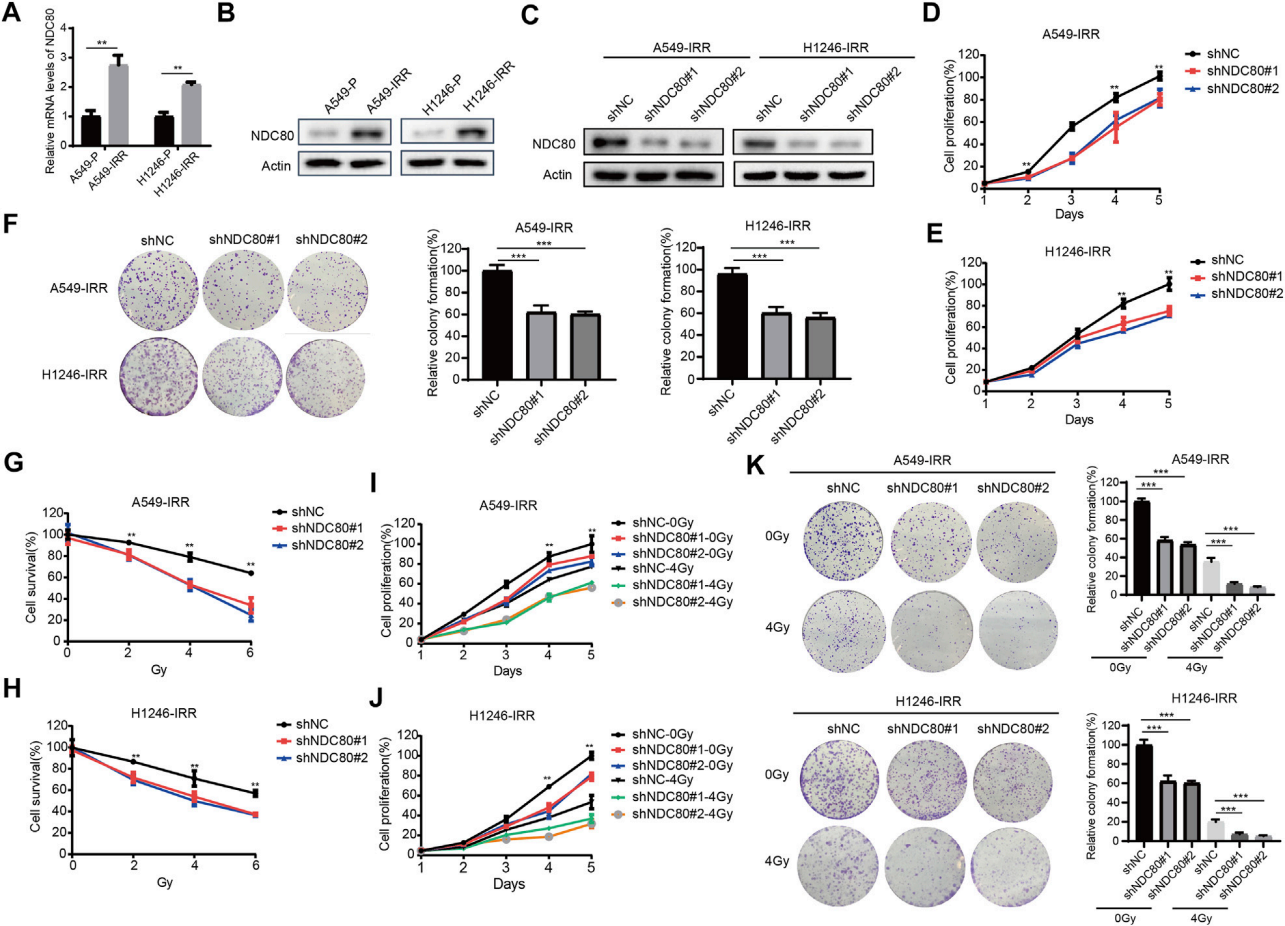
Knockdown of NDC80 increases the radiosensitivity of lung cancer cells. **(A,B)** The mRNA **(A)** and protein **(B)** expression of NDC80 was examined in A549 and H1246 parental and IR-resistant cells. **(C)** After the stable knockdown of NDC80 in A549-IRR and H1246-IRR cells, the protein expression of NDC80 was analyzed using the Western blot assay. **(D,E)** Cell proliferation was examined in A549-IRR **(D)** and H1246-IRR **(E)** cells with shNC or shNDC80. **(F)** Colony formation was analyzed in A549-IRR and H1246-IRR cells via the stable reduction of NDC80. **(G–H)** Dose-dependent cell viability (exposure to 0,2,4,6 Gy) was examined in A549-IRR **(G)** and H1246-IRR cells **(H)** with NDC80 knockdown. **(I–K)** A549-IRR and H1246-IRR cells with shNC or shNDC80 were exposed to irradiation at a dose of 4 Gy and evaluated via CCK-8 assays **(I,J)** and colony formation assays **(K)**. *p < 0.05; **p < 0.01; ***p < 0.001.

To further investigate whether NDC80 was involved in radioresistance development in lung cancer cells, we determined the expression of NDC80 in A549 and H1246 parental and IR-resistant cells (Chen et al., 2021). The radioresistance feature of H1246-IRR was identified in Supplementary Figure S2. Through qPCR and western blot assays, mRNA and protein levels of NDC80 were found to be notably upregulated in both A549-IRR and H1246-IRR cells, compared to their parental cells (Figures 4A,B). Subsequently, we established stably NDC80-depleted A549-IRR and H1246-IRR cells with two shRNAs (Figure 4C). Cell proliferation was significantly inhibited in A549-IRR and H1246-IRR cells after the knockdown of NDC80 (Figures 4D,E). Likewise, colony formation was notably reduced for shNDC80, compared to that observed for shNC (Figure 4F). To confirm the effects of NDC80 on radio-resistance in lung cancer cells, A549-IRR and H1246-IRR cells exhibiting stable NDC80 reduction were treated with IR. The results showed that the downregulation of NDC80 resulted in a dose-dependent inhibition of cell survival after treatment with 0, 2, 4, and 6 Gy IR (Figures 4G,H). Moreover, the knockdown of NDC80 enhanced the radiosensitivity of A549-IRR and H1246-IRR cells, in which the cell viability and colony formation were decreased by more than 50% after exposure to 4 Gy IR (Figures 4I–K). Collectively, these results suggest that the downregulation of NDC80 improved the sensitivity of lung cancer cells to IR.

### The role of NDC80 in the regulation of autophagy in lung cancer patients

**FIGURE 5:**
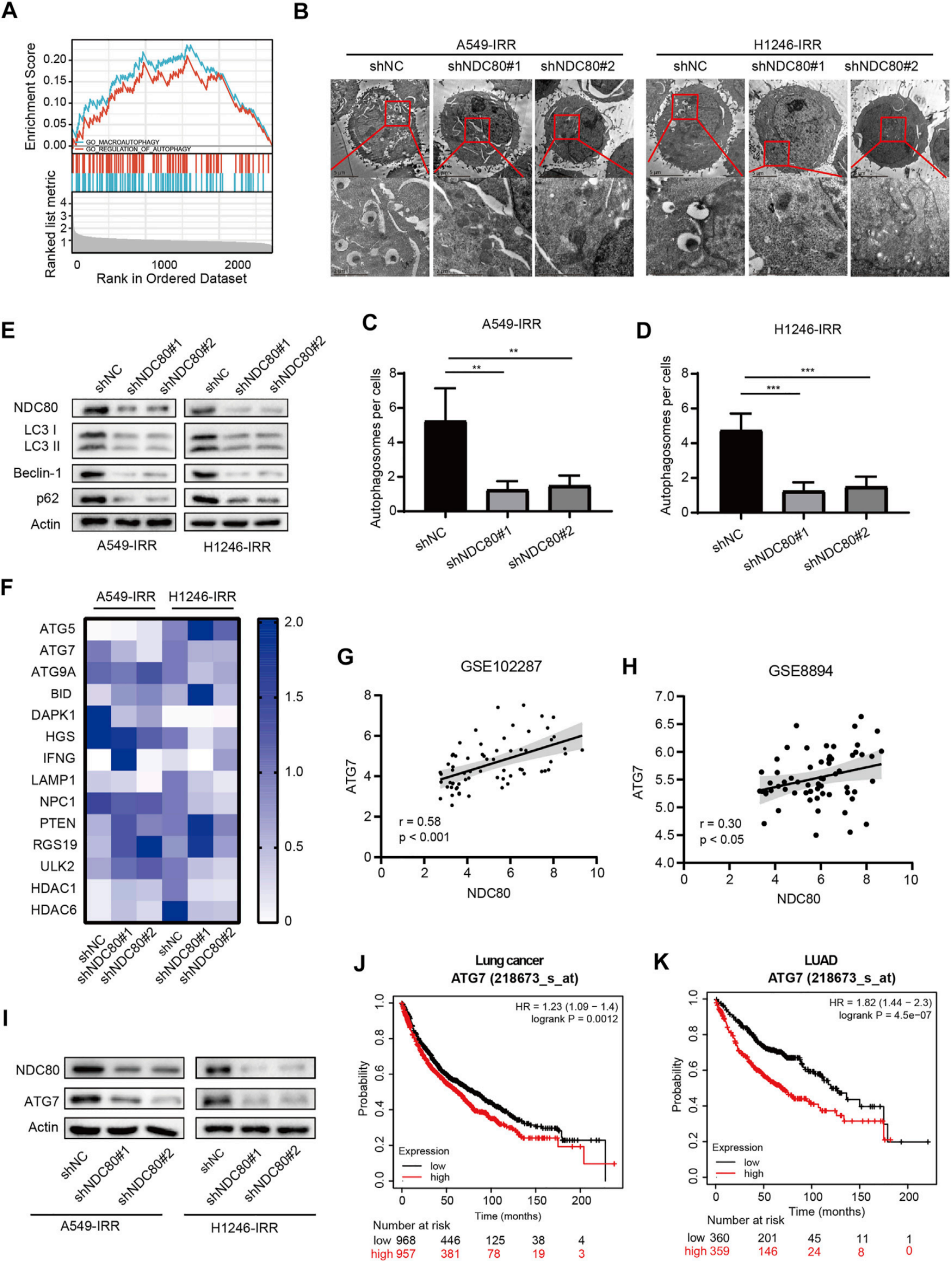
NDC80 regulates autophagy in lung cancer. **(A)**Analysis of the correlation between NDC80 and autophagy by GSEA analyses. **(B–D)** Autophagosome formation was detected by TEM analysis in A549-IRR and H1246-IRR cells with NDC80 knockdown. **(E)** After the knockdown of NDC80 in A549-IRR and H1246-IRR cells, LC3 II, Beclin-1, and p62 protein levels were detected. **(F)** The heatmap shows the mRNA levels of 14 autophagy-relevant genes identified in A549-IRR and H1246-IRR cells. **(G,H)** Validation of the correlation between NDC80 and ATG7 in two datasets, i.e., GSE102287 **(G)** and GSE8894 **(H)** in the GEO database. **(I)** Analysis of the protein expression level of ATG7 in NDC80-reduced A549-IRR and H1246-IRR cells. **(J,K)** Analysis of the prognostic value of ATG7 in lung cancer **(J)** and LUAD **(K)**, through Kaplan-Meier analysis. **p < 0.01; ***p < 0.001.

Autophagy is a self-renewal process that devours cellular proteins and organelles, and was proposed as a protective mechanism for tumor cell survival in radiotherapy (Levy et al., 2017). In the present study, we performed GSEA using the information from iTRAQ analysis, and confirmed that autophagy might be affected by NDC80-affected biological functions (Figure 5A). Next, we assessed the NDC80-mediated autophagy in IR-resistant NSCLC cells. TEM results showed that a number of autophagosomes exhibited a significant tendency to decrease in A549-IRR and H1246-IRR cells with NDC80 depletion (Figures 5B–D). We then determined the protein markers involved in the formation of autophagosomes, including Beclin-1, p62, and LC3 II *in vitro*. The protein expression levels of LC3-II, p62, and Beclin-1 were reduced in shNDC80, compared to shNC in A549-IRR and H1246-IRR cells (Figure 5E). To further unveil the molecular mechanisms underlying NDC80-regulated autophagy in lung cancer, the results from iTRAQ analysis were implemented to screen autophagic regulators. As shown in Supplementary Figure S3, autophagic regulators, including 12 upregulated and 2 downregulated proteins, were selected for further identification, according to their expression levels in iTRAQ analysis. Using the qPCR array, we examined the mRNA expression of these candidates in A549-IRR and H1246-IRR cells with stable NDC80 knockdown. The results showed that only ATG7 mRNA expression was markedly downregulated, after values were filtered using the criterion of foldchange >1.5 (Figure 5F). It is known that ATG7 acts as an E1-like activating enzyme and plays a vital role in mediating autophagy (Zhou et al., 2020). Therefore, we used two GEO datasets, GSE102287 and GSE8894, of which GSE102287 was comprised of NSCLC samples and GSE8894 was comprised of LUAD samples, for identifying the correlation between NDC80 and ATG7. The results showed that the expression of NDC80 was positively associated with the levels of ATG7 in lung cancer, using the GSE102287 and GSE8894 datasets (Figures 5G,H). Furthermore, we detected the protein expression level of ATG7 in NDC80-depleted IR-resistant NSCLC cells. The level of ATG7 was notably decreased, and this was accompanied by a reduction in the NDC80 levels in A549-IRR and H1246-IRR cells (Figure 5I). In addition, using Kaplan-Meier analysis, we found that patients with high expression levels of ATG7, and especially those with LUAD, showed unfavorable survival in lung cancers (Figures 5J,K).

### Identification of correlation between expression of NDC80 and ATG7 by immunohistochemistry

**FIGURE 6:**
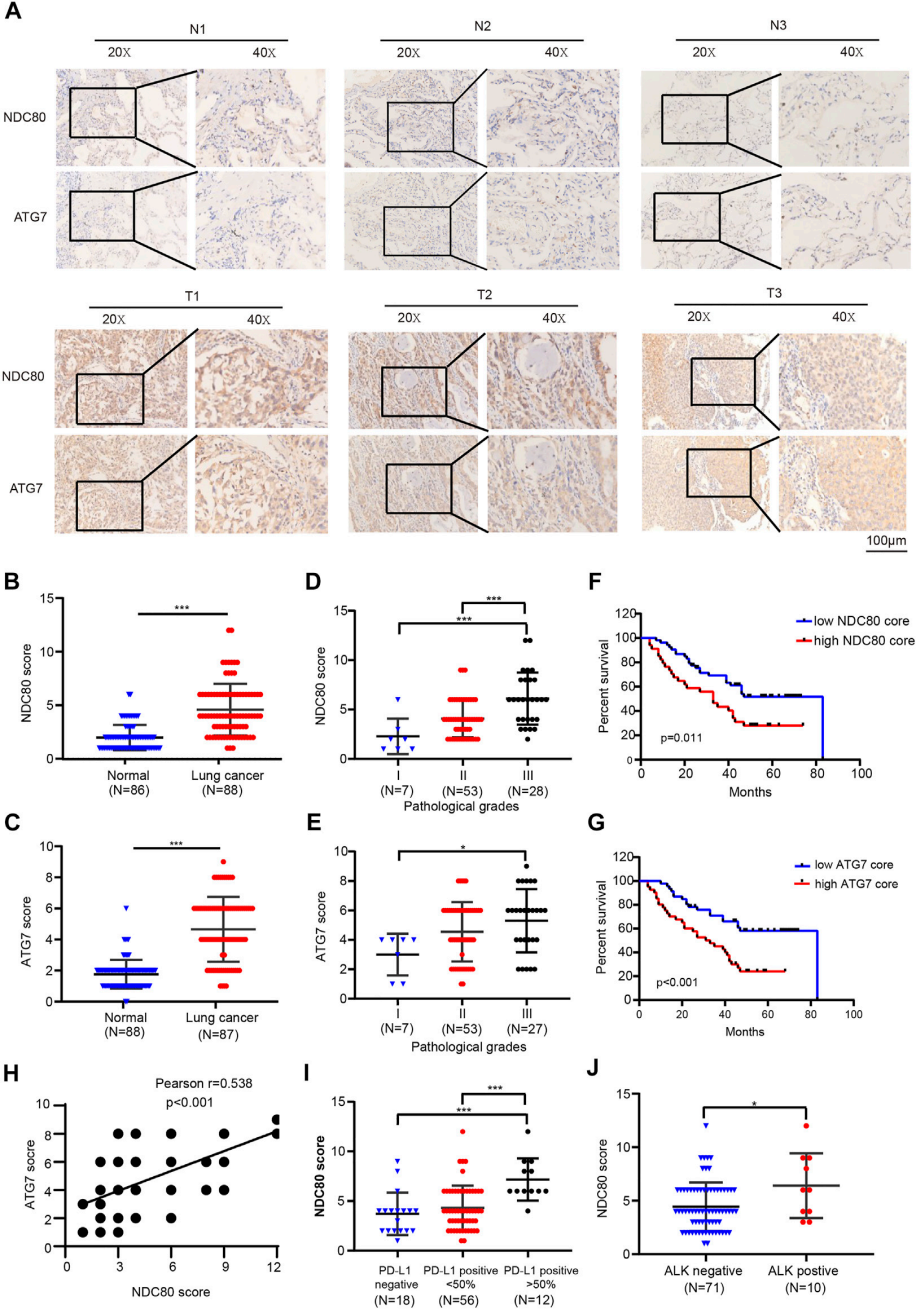
Identification of the correlation between the expression of NDC80 and ATG7 by IHC. **(A–C)** Determination of the expression levels of NDC80 and ATG7 in LUAD and healthy lung specimens by IHC analysis. **(D,E)** The comparison of NDC80 **(D)** and ATG7(E) expression in pathological stages. **(F,G)** The OS analysis of high- and low-expression levels of NDC80 **(F)** and ATG7(G). **(H)** The correlation between the expression of NDC80 and ATG7. **(I,J)** The correlation of NDC80 with PD-L1 expression **(I)** and ALK status **(J)**. *p < 0.05; ***p < 0.001.

The above findings indicated that the oncogenic effects of NDC80 might induce autophagy by mediating ATG7 in IR-resistant cells of lung cancer. Thus, we further validated the tumorigenic role of NDC80 and ATG7 in LUAD samples by IHC assays. As shown in Figures 6A–C, the staining intensity of NDC80 and ATG7 was stronger in LUAD samples than in healthy specimens. Furthermore, the expression of NDC80 in Grade III pathological stage specimens was higher than that in Grade II and Grade I, respectively (Figure 6D). Consistently, the level of ATG7 was significantly increased in Grade III, as compared to that in Grade I (Figure 6E). Kaplan-Meier survival analysis suggested that LUAD patients with higher NDC80 or ATG7 expression levels had a poorer prognosis than those with lower expression levels (Figures 6F,G). We also found that the increased expression of NDC80 was concomitant with the elevated level of ATG7 (Figure 6H). Furthermore, targeted therapies for several oncogenic alterations, such as PD-L1-positive expression and ALK translocation have been incorporated during the clinical treatment of lung cancers (Imyanitov et al., 2021), Interestingly, higher expression levels of NDC80 were observed if PD-L1>50% LUAD than if it was <50% PD-LI and negative tissues (Figure 6I). In addition, NDC80 expression was significantly enhanced in patients who were ALK-positive (Figure 6J). All these results suggest that NDC80 might become a potential biomarker for the treatment of lung cancer.

## Discussion

In this study, we showed that NDC80 served as a prognostic indicator of lung cancer. Furthermore, we found that NDC80 was associated with the expression of several diagnostic and therapeutic biomarkers of lung cancer, including TP53, PD-L1, and ALK. Moreover, we verified that NDC80 promoted cell growth and radioresistance in IR-resistant lung cancer cells by targeting autophagy. These findings suggest that the targeting of NDC80 might be a potential approach for overcoming the development of resistance in radioresistant NSCLCs.

Autophagy is a crucial and controversial process that helps maintain intracellular homeostasis as it plays dual functions in cell survival and death. Hence, the molecular mechanisms underlying autophagy-regulated radioresistance remain blurred and complicated. Several studies have shown that the blockade of autophagy signaling, including PI3K/mTOR, AMPK, and c-Jun signaling, has the potential to improve radiotherapeutic efficacy (Chaachouay et al., 2015; Zhang et al., 2016). In addition, we previously found that in IR-resistant NSCLC cells, LC3 II, Beclin-1, and p62 levels were notably higher than that of paired parental cells, while the knockdown of Cav1 decreased these protein levels and increased radiosensitivity (Chen et al., 2021). Our present study has revealed for the first time that NDC80-mediated has a cytoprotective role in autophagy in IR-resistant NSCLC cells. We observed that the downregulation of NDC80 in IR-resistant cells reduced the expression levels of LC3 II, Beclin-1, and p62 and the formation of autophagosomes. So far, there have been few reports on the relationship between NDC80 and autophagy. A study reported that NDC80, as a component of the KMN (KNL-1/Mis12/Ndc80) complex, could indirectly interact with Beclin-1, which is essential for kinetochore assembly in Hela cells (Fremont et al., 2013). This study shows that NDC80 could influence cell survival by affecting autophagy. Altogether, these data explain the mechanism by which NDC80 overcomes resistance to IR through pro-survival autophagic functions.

Furthermore, we elucidated that NDC80 regulates autophagy by targeting ATG7 in IR-resistant NSCLC cells. ATG7, an E1-like enzyme, is required for the conjugation of Atg12 to Atg5, which was coupled with phosphatidylethanolamine to LC3 (Collier et al., 2021). ATG7 has been observed to promote tumor development, since the knockdown of ATG7 could inhibit the self-renewal and invasion of stem-like lung cancer cells (Zhou et al., 2021). Furthermore, ATG7 was reported to be overexpressed in lung cancer with cisplatin treatment, which causes resistance to apoptosis through cisplatin-induced hydroxyl radicals (Sumkhemthong et al., 2021). As for radiotherapy, ATG7 deficiency could sensitize cancer cells to IR (Schaaf et al., 2015). Moreover, the accumulation of autophagosomes with concomitantly elevated mRNA levels of ATG7 leads to radiation resistance in breast cancer under hypoxic exposure (He et al., 2012). These studies suggest that the effects of ATG7 on radiation treatment may be determined by the types of cancers and conditions. In the present study, we observed that the knockdown of NDC80 attenuated ATG7 expression in IR-resistant cells. Meanwhile, ATG7 was upregulated to a greater extent in LUAD samples than in normal tissues, and was positively correlated with the NDC80 expression level. These results indicate that the downregulation of NDC80 alleviates IR-resistant features in NSCLC cells through the regulation of ATG7-related autophagy.

In summary, in this study, we identified that NDC80 might be a diagnostic and prognostic indicator in lung cancer. Furthermore, elevated NDC80 expression was detected in IR-resistant NSCLC cells, and was found to induce radiation resistance. Our findings provide novel insights into the effect of NDC80 on radioresistance in cancer cells, and suggest that NDC80 could serve as a drug target for improving radiosensitivity.

## Data Availability

The datasets presented in this study can be found in online repositories. The names of the repository/repositories and accession number(s) can be found in the article/Supplementary Material.
