# Supplementary figures and images for: Upregulation of nuclear division cycle 80 contributes to therapeutic resistance via the promotion of autophagy-related protein-7-dependent autophagy in lung cancer
Source: Front Pharmacol. 2022 Aug 29;13:985601. doi: 10.3389/fphar.2022.985601 (PMC9465246; doi:10.3389/fphar.2022.985601)

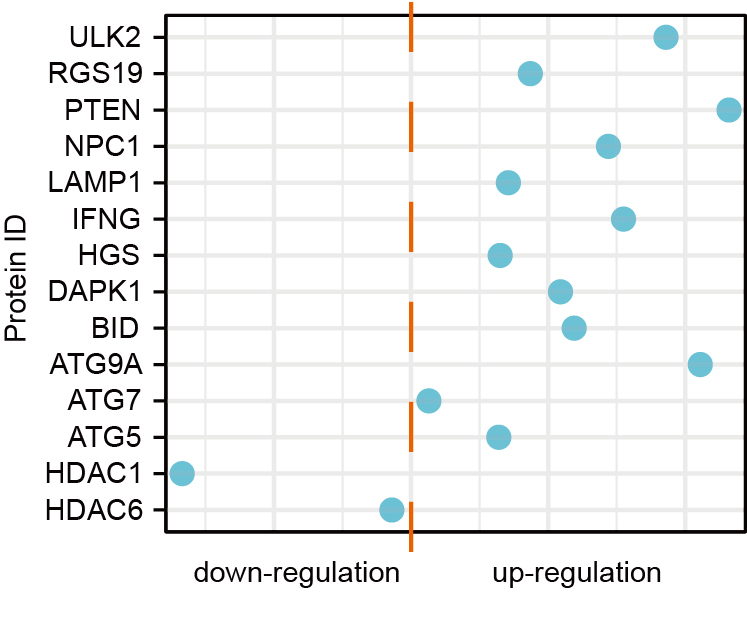

Supplement: Supplementary file 2 [file Image3.JPEG]

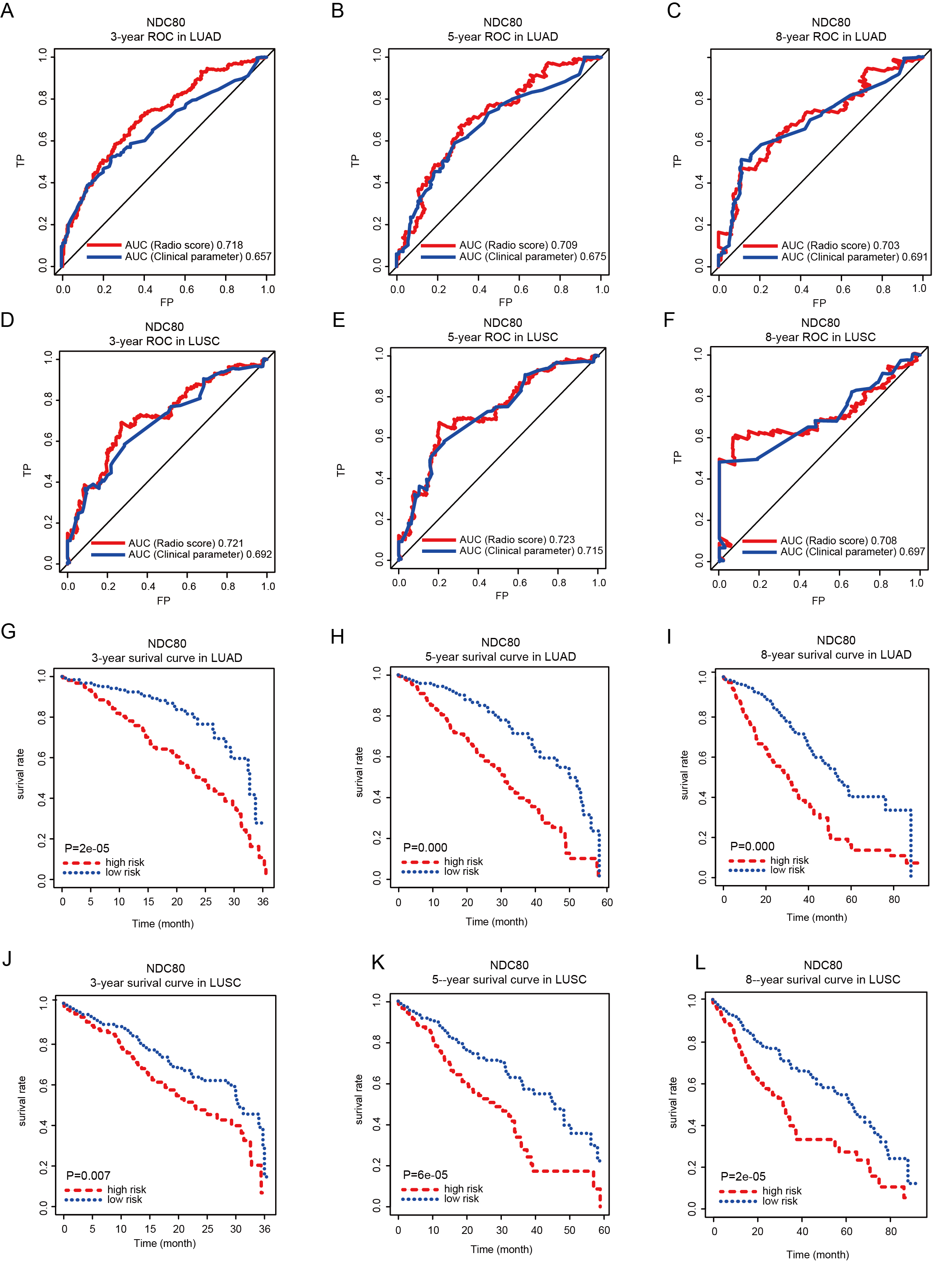

Supplement: Supplementary file 4 [file Image1.JPEG]

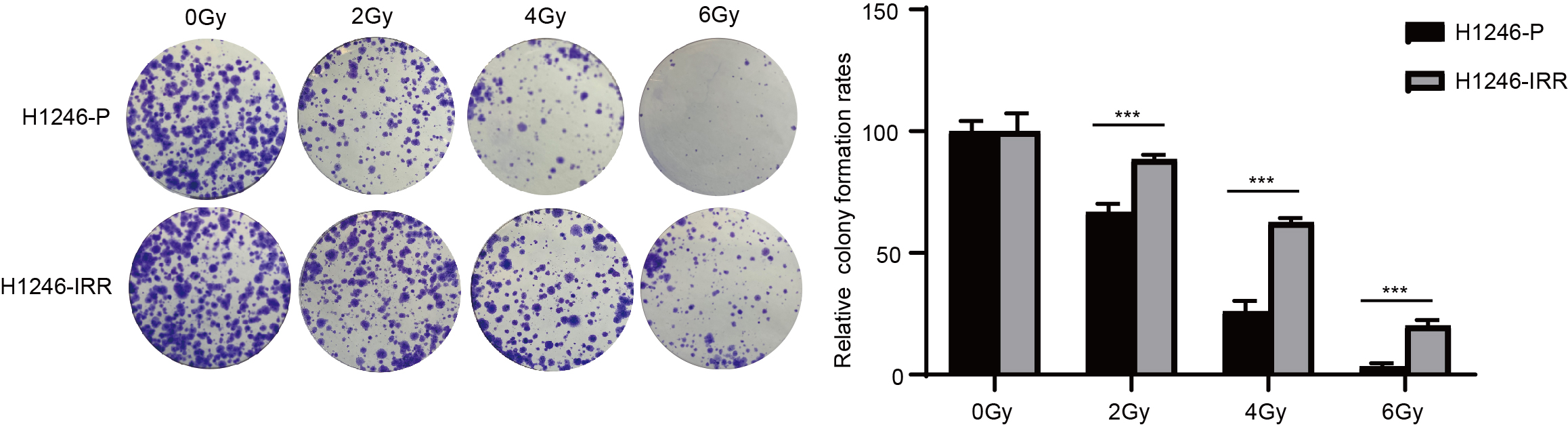

Supplement: Supplementary file 5 [file Image2.JPEG]
