# Supplementary material for: Upregulation of nuclear division cycle 80 contributes to therapeutic resistance via the promotion of autophagy-related protein-7-dependent autophagy in lung cancer
Source: Front Pharmacol. 2022 Aug 29;13:985601. doi: 10.3389/fphar.2022.985601 (PMC9465246; doi:10.3389/fphar.2022.985601)
Supplement: Supplementary file 7 [file DataSheet1.docx]

Supplementary Material

# Supplementary Figures and Tables

## Supplementary Figures

**Supplementary Figure S1.** **Survival analysis of NDC80 in LUAD and LUSC datasets from the TCGA database**

(A-F) Use of time-dependent ROC curves for verifying the prognostic role of NDC80 in two TCGA datasets, including LUAD (A-C) and LUSC (D-F) datasets. (G-L) Use of survival curves to determine the OS of patients in the low-risk and high-risk groups in the TCGA datasets of LUAD (G-I) and LUSC (J-L).

**Supplementary Figure S2. Confirmation of the IR-resistant phenotypes of H1246-IRR cells**

**Supplementary Figure S3. The 14 autophagy-related protein in iTRAQ analysis**

## Supplementary tables

**Supplementary Table S1. The primer sequence of 14 autophagy-related genes**

**Supplementary Table S2. iTRAQ analysis of A549-IRR vs. A549-P**

**Supplementary Table S3. The DEGs in LUAD and LUSC from datasets of TCGA**
